# Soya saponin improves egg-laying performance and immune function of laying hens

**DOI:** 10.1186/s40104-021-00647-2

**Published:** 2022-01-05

**Authors:** Peng Li, Yizhu Zhao, Shaojia Yan, Bocheng Song, Yongfa Liu, Mingkun Gao, Dazhi Tang, Yuming Guo

**Affiliations:** State Key Laboratory of Animal Nutrition, China Agricultural University, Beijing, 100193 China

**Keywords:** Egg-laying performance, Immune function, Laying hens, Soya saponin

## Abstract

**Background:**

Soya saponin (SS), an active compound in soybean meals, has been widely studied in the medical field. However, it was considered as an anti-nutritional factor in poultry diets. The objective of this experiment was to measure the effects of dietary SS using three dietary treatments on egg-laying performance and immune function of laying hens. Birds were fed a low soybean meal basal diet (CON), a low-SS diet (50 SS) containing 50 mg/kg SS, or a high-SS diet (500 SS) containing 500 mg/kg SS for 10 weeks. At the end of the 5th and 10th week of the trial, samples were collected for analysis.

**Results:**

Results showed that with 50 mg/kg SS supplementation, the egg production rate, feed conversion ratio (FCR), and eggshell quality tended to be improved. Serum follicle stimulating hormone (FSH) and Interleukin-4 (IL-4) levels were also elevated as well as the peripheral blood LPS stimulation index, the proportion of B lymphocytes, and antibody titer of bovine serum albumin (BSA). We also found that mRNA levels of follicle stimulating hormone receptor (*FSHR)* in ovarian, nuclear transcription factor kappa B (*NF-κB),* Transforming growth factor *(TGF-β*) and interferon γ (*IFN-γ*) in spleen were up-regulated at the end of the trial. Additionally, dietary 50 mg/kg SS improved the ileal flora via up-regulating the relative abundance of *Lactobacillus, Romboutsia* and *Lactobacillus delbrueckii*. Although the immune related indicators were improved with 500 mg/kg SS supplemented, it seemed to have a negative influence on the laying-performance. Specifically, serum alanine aminotransferase (ALT), alkaline phosphatase (ALP), and the ratio of IFN-γ to IL-4 were increased in the 500 SS group at the end of the trial. The mRNA levels of gonadotropin releasing hormone 1 (*GnRH1*) in Hypothalamus, the estrogen related receptor (*ERR*) in ovaries were downregulated as well as the egg production rate during the trial with 500 mg/kg SS supplemented.

**Conclusions:**

The egg production performance was improved by dietary supplemented with 50 mg/kg SS via increasing ovarian *FSHR* transcription level and serum estrogen level. A beneficial shift in intestinal microflora was recorded, and the immune function of laying hens was also improved with 50 mg/kg SS supplementation. Surprisingly, the long-term supplementation of 500 mg/kg SS exerted a negative impact on the laying performance and physiological functions of the liver of laying hens.

**Supplementary Information:**

The online version contains supplementary material available at 10.1186/s40104-021-00647-2.

## Introduction

Soya saponin (SS) is a pentacyclic triterpenoid naturally occurring compound in soybeans. In the past, SS was considered as an anti-nutritional factor in soybean meals for poultry. Nevertheless, it has been widely studied in recent years because of its immunomodulatory effects. SS is composed of soybean sapogen and some glycosides, uronic acid, etc. Due to the difference in glycosides, SS is divided into four different types: A, B, E, DDMP [1].

The major biological roles of SS have been reported to be associated with inhibiting the production of inflammatory factors via regulating toll-like receptor 4 (*TLR4*)-nuclear factor kappa beta (*NF-κB*) and phosphatidylinositol 3-kinase (*PI3K*)-protein kinase B (*PKB*)-*NF-κB* pathway [2, 3]. Likewise, it also reduced the production of inflammatory factors by activating peroxisome proliferator-activated receptor-γ (*PPAR-γ*) [4]. Some findings suggested its role in down-regulating the production of reactive oxygen species (ROS) to relieve oxidative stress via regulating NF-E2-related factor 2 (*Nrf-2*)-antioxidant response element (*ARE*) pathway [5]. SS could also relieve osteoporosis in mice by activating Smads molecules to promote the transcription of runt-related transcription factor 2 (*Runx2*) and osterix (*Osx*) genes [6, 7]. A number of studies reported the beneficial effects of SS supplementation to the diets. To illustrate, the antibody titer of Newcastle disease in broiler serum was elevated without affecting the production performance with 5 mg/kg SS oral treatment [8]. Study also suggested there were no negative effects on its performance, intestinal morphology and organ index with 20 mg/kg SS supplemented to the diet of mice [9]. Another study suggested that dietary 20 mg/kg SS alleviated 2,4-dinitro fluoro benzene (DNFB)-induced intestinal microflora imbalance in mice [10]. Most of these studies were done in the medical field.

*In vitro* studies showed that the proliferation of *Staphylococcus aureus* and *Escherichia coli* were inhibited by SS [11, 12]. SS could also alleviate allergic skin reactions in mice via improving the intestinal microflora [10]. Study suggested that the products of SS fermented by *Lactobacillus pentosus* could alleviate memory impairment in mice [13]. SS has a similar molecular structure to ginsenosides. It was demonstrated that ginsenoside could promoted proliferation of ovarian cells from chicken through PKC activation and up-regulated cyclin gene expression, and alleviated ovaries dysfunction caused by excessive pregnant mare serum gonadotrophin (PMSG) [14–16]. Study also suggested that ginsenosides inhibited the apoptosis of ovarian granulosa cells from mice, and inhibited the secretion of inflammatory factors [17]. We hypothesized that it could be interesting to explore the effect of SS on reproductive system.

To our knowledge, there is almost no research about the influence of SS on poultry, and the study on the interaction between SS and gut microbes is extremely limited. We hypothesized that dietary an appropriate dose of SS might improve laying performance, immune function, and intestinal microflora of laying hens.

## Materials and method

All procedures adapted for the experiment were approved by the Animal Ethics Committee of China Agricultural University, Beijing, China. The animal welfare number was AW92601202–1-1.

### Experimental design and animal management

A total of 270, 21-week-old Hy-line grey layer hens were housed in a conventional cage in a closed house. The cages were arranged in 3 tiers with 5 cages per tier and 3 birds per cage. The common standard diet was fed for 1 week pre-feeding period. After the pre-feeding period, 270 22-week-old Hy-line grey hens were randomly divided into three treatment groups according to the principle of uniform egg production rate (47 ± 0.02%) and similar body weight (1470 ± 10 g). Control group (basal diet with low soybean meal, the diet formula was showed in Table 1), 50 SS group (Basal diet supplemented with 50 mg/kg SS), 500 SS group (Basal diet supplemented with 500 mg/kg SS). There were 6 replicates per treatment and 15 birds per replicate. The test SS was purchased from Xi’an Tongze Biotechnology Co., Ltd. (Shaanxi, China; total SS content was 45.1%). The experimental diets were fed for 10 weeks. Eggs were collected and weighed once at 16:00 h every day. The temperature of laying hen room was controlled at 25 ± 3 °C, besides, 16 h light: 8 h dark lighting program was used. Egg quality was measured every 3 weeks while egg production and feed efficiency were calculated every week. At the end of the 5th week, six laying hens with uniform body weight and egg production rate from each group were selected to collect blood from the wing vein, and then to harvest serum and extract lymphocytes for subsequent analyses. At the same time, those birds were anesthetized with 50 mg/kg BW of sodium pentobarbital, and then slaughtered to obtain ovaries, fallopian tubes and ileal chyme for further analyses. At the 5th and 7th week of the trial, eight birds from each group were selected for intramuscular injection of 1 mL of 1% bovine serum albumin (BSA, Roche 738,328, Switzerland) solution. 7 and 14 days after BSA injection, blood was collected from the wing vein to harvest serum for testing. At the end of the trial, eight birds from each group were selected to collect blood, and then anesthetized and killed to collect ovaries, fallopian tubes, hypothalamus, liver and spleen for further testing.

**Table 1:** Test diet composition and nutrition level of air-dry basis

Ingredients	Contents, %	Nutritional parameters^c^	Levels
Corn, 7.8% pro	67.55	ME, MC/kg	2.701
Dephenolized cottonseed protein, 50%	14.00	Crude protein, %	16.530
Limestone powder	8.154	Calcium, %	3.630
Corn gluten meal, 51.3%	5.00	Total phosphorus, %	0.764
Soybean meal, 48%	2.00	Available phosphorus, %	0.434
Ca (HCO_3_)_2_	1.86	Methionine + Cystine, %	0.681
NaCl	0.35	Lysine, %	0.791
Trace minerals^b^	0.30	Methionine, %	0.408
L-Lysine HCl, 78%	0.25	Threonine, %	0.576
DL-Methionine	0.12	Tryptophan, %	0.164
Choline chloride, 50%	0.12	Digestible Lysine, %	0.725
Tryptophan	0.02	Digestible Methionine, %	0.386
Multi-vitamins^a^	0.03	Digestible Threonine, %	0.505
Sandaquin	0.03	Digestible Tryptophan, %	0.148
Phytase	0.016		
Zeolite powder	0.2		
Total	100		

The diet was formulated with reference to NY /T33–2004. The content of SS in our SS additives was 45.1%, and the actual measured values of SS in each group of diets are as follows. The content of SS in the diet of the control group was 9.5 mg/kg. The contents of SS in the 50 SS and 500 SS groups were 31.3 and 275.7 mg/kg, respectively. ^a^Vitamin premix (provided per kilogram of feed) the following substances: vitamin A, 12,500 IU; vitamin D_3_, 2500 IU; vitamin K_3_, 2.65 mg; vitamin B_1_, 2 mg; vitamin B_2_, 6 mg; vitamin B_12_, 0.025 mg; vitamin E, 30 IU; biotin, 0.0325 mg; folic acid, 1.25 mg; pantothenic acid, 12 mg; niacin, 50 mg. ^b^Trace element premix (provided per kilogram of feed) the following substances: copper, 8 mg; zinc, 75 mg; iron, 80 mg; manganese, 100 mg; selenium, 0.15 mg; iodine, 0.35 mg. ^c^Calculated value based on the analysis of experimental diets

### Determination of production performance and egg quality

Egg production rate, feed consumption and feed to egg production ratio was calculated. Egg production rate (%) = total number of eggs laid during the statistical period / (number of housed hens × number of statistical days) × 100%. Average egg production rate during the test period (%) = total number of eggs laid during the test period / (number of hens housed × total days of the test) × 100%. Feed-to-egg ratio (FCR) = total material consumption during the test / total egg weight during the test.

All eggs from each treatment within 24 h were collected to measure eggshell thickness, eggshell strength, haugh units, albumen height and egg yolk color. The egg quality tester DET-6000 (NABEL Co., Ltd., Japan) was used to measure the eggshell strength (kg/cm^2^) and egg yolk color after weighing the eggs. Briefly, the eggs were placed vertically on the eggshell strength tester, with the blunt end up, to measure the pressure on the eggshell surface per unit area. Albumen height was determined with the albumen height measuring instrument KIYA-818B (SEISAKUSHO, LTD), and then the Haugh unit was calculated according to the formula: Haugh Unit = 100 lg(H − 1.7 W^0.37^ + 7.57), where H = the albumen height (mm) and W = the egg weight (g). The thickness of the eggshell was measured with a micrometer. Specifically, after removing the shell membrane from the eggshell, the shell thickness of the blunt end, the middle and the sharp end of the egg were measured to calculate the average of the three. The average value was in millimeters, accurate to 0.01 mm.

### Organ index and liver morphology

The ovaries, oviducts, liver and spleen were weighted with an electronic balance (accurate 0.01 g). A ruler (accurate 0.01 mm) was used to measure the length of ovary, the total length of the oviduct, the length of the magnum and the shell gland. At the end of the trial, liver tissue samples about 1 cm^2^ were collected and then suspend it into 4% paraformaldehyde solution. Liver sections were made and then stained with eosin-hematoxylin (HE stain). The Olymps BX-41TF microscope was used to observe the infiltration state of inflammatory cells in liver slices. The magnification was 400 times.

### Determination of serum hormone levels, immune indexes and biochemical indexes

Blood was collected from the wing vein, and then serum was harvested by centrifugation at 3000 rpm and 4 °C for 15 min. The contents of Follicle stimulating hormone (FSH), Luteinizing hormone (LH), Estradiol (E2) and Progesterone (P4) in serum were detected by radioimmunoassay using commercial kit (from Beijing Northern Biotechnology Institute, Beijing, China). The detection coefficient of variation was less than 10%. The levels of immunoglobulin G, immunoglobulin A and immunoglobulin M in the serum was detected according to the manufacturer’s guidelines of Elisa kits (Beijing Solarbio Biotechnology Co., Ltd., China). ELISA kits (IDEXX laboratories lnc., Weatbrook, Maine, USA) were used to determine the levels of interleukin-2 (IL-2), IL-6, IL-4 and interferon-γ (IFN-γ) in the serum at the end of 5th and 10th week. The ratio of IFN-γ to IL-4 was also calculated. Following the manufacturer’s guidelines (Nanjing Jian cheng Biotechnology Co., Ltd., China), an automatic biochemical analyzer (Unicel DXC800, Beckman Coulter, USA) was used to analyze the levels of total serum protein (TP), albumin (ALB), alanine aminotransferase (ALT), aspartate aminotransferase (AST), alkaline phosphatase (ALP) and glucose (GLU) at the end of the trial.

### Determination of serum BSA antibody titer

According to the previous [18], the indirect ELISA method was used to measure the anti-BSA antibody titer in the serum. Briefly, BSA at a concentration of 2 mg/mL was used to coat 96-well plates and incubated overnight at 4 °C. Subsequently, 0.01 mol/L PBS-Tween (pH 7.4, 0.05% Tween 20) was used to wash the 96-well plate for 5 times, and the serum samples were added to the washed 96-well plate and incubated at 37 °C for 1.5 h. Peroxidase-labeled chicken polyclonal IgG antibody (Bethyl L aboratories, Inc. US.) was added to the washed 96-well plate to specifically bind to BSA. After that, 100 μL of secondary antibody was added to the washed 96-well plate and incubated at 37 °C for 30 min. After washing, 100 μL of 0.05% tetramethyl acridine was added to the reaction well, incubated at room temperature in the dark for 30 min, and finally terminated with 2 mol/L sulfuric acid. An automatic microplate reader (SepctraMax i3x Platform, Molecular Devices, LLC, Australia) was used to read the absorbance value.

### Peripheral blood lymphocyte ratio and stimulation index

According to the method of Fan et al. [19], heparinized blood from the wing vein were diluted with sterile calcium- and magnesium-free Hank’s balanced salt solution (CMF-HBSS, Sigma, St. Louis, MO, USA) at a 1:1 ratio and placed on ice. The diluted samples were carefully transferred into a tube added with an equal volume of Ficoll lymphocyte separation medium (Histopague-1077, Tianjin HaoYang Biological Manufacture Co., Ltd., China) to form a distinct layer above the Ficoll. The mixture was centrifuged at 400 × *g* for 30 min at room temperature. The centrifuged mixture was divided into three layers, and the white flocculent material in the middle was transferred into a clean tube. The RPMI 1640 (Invitrogen Corp., Grand Island, NY, USA) incomplete culture medium was used to wash the lymphocyte suspension for 3 times, and then the lymphocyte was resuspended into 2 mL of RPMI 1640 complete culture medium. The cell counter was used to make sure the live cell concentration as 1 × 10^7^ cell/mL.

The following primary monoclonal antibodies were diluted in PBS (pH 7.2): IgG1κ mouse anti-chicken-Bu-1-PB-labelled antibody (8395–26), IgG1κ mouse anti-chicken-Monocyte/Macrophage-PE-labelled antibody (8420–09), IgG1κ mouse anti-chicken-CD3-APC-labelled antibody (8200–11), and IgG1κ mouse anti-chicken- CD45-PE- labelled antibody (8270–09) (Southern Biotechnology Associates Inc., Birmingham, AL, USA). A 1-mL eppendorf tube was stained with 25 μL of diluted primary monoclonal antibody (1:100 dilution) and the negative isotype control IgG, and then a volume of 100 μL of lymphocytes extracted above (2 × 10^6^ cells) was added into this tube for incubation about 45 min at room temperature. After that, the cold PBS was used to wash the cells twice for removing the unbound primary antibodies. A total of 300 μL of hemolysin solution diluted in PBS (1:25) was added to each tube. Finally, the cells were washed twice and adjusted to a final volume of 500 μL. Four-color flow cytometric analysis was conducted using a Navios EX flow cytometer with 10 colors (Beckman Coulter Corp., Fullerton, CA, USA) at Xi-Yuan Traditional Chinese Medicine Hospital, Chinese Academy of Medicine Science, China. The percentages of CD3^+^ T cells, Monocytes/Macrophages, and Bu-1 were subsequently calculated. The result was expressed as a percentage.

A 3-(4,5-dimethylthiazol)-2,5-diphenyltetrazolium bromide (MTT, Sigma Chemical Co., St. Louis, MO, USA) method was used to determine the peripheral blood lymphocyte proliferation response [20]. The stimulus index of concanavalin A (Con A, 45 μg/mL) on T cells and lipopolysaccharide (LPS, 25 μg/mL) on B cells were analyzed. The results were expressed in terms of the stimulus index (SI) value. Both Con A and LPS were purchased from Sigma-Aldrich.

### Gene expression measurement and analysis

Hypothalamus, ovarian, liver, and spleen samples were collected and placed in RNase-free Centrifuge tube, and then the samples were quickly placed in liquid nitrogen. Taking 100 mg tissue sample into 1-mL Trizol (Invitrogen Life Technologies, Carlsbad, CA, USA), Total RNA isolation, quantification, cDNA synthesis, and real-time PCR were carried out as previously described by Zhao et al. [21]. Total RNA was quantified by using the NanoDrop® ND-2000 UV-VIS spectrophotometer (Thermo Scientific, Wilmington, DE, USA) at an OD of 260 nm, and the purity was assessed by determining the OD260/OD280 ratio. All the samples had an OD260/OD280 ratio above 1.8, corresponding to 90–100% pure nucleic acids. Meanwhile, the integrity of RNA in each sample was assessed using 1% denatured agarose gel electrophoresis. RNA was used for RT-PCR analysis when it had a 28 S/18 S rRNA ratio ≥ 1.8. Total RNA was reverse-transcribed using the PrimeScript® RT reagent Kit with gDNA Eraser (Takara, Dalian, China) according to the manufacturer’s instruction. cDNA was synthesized and stored at − 80 °C until use. The RT-PCR analysis of gene expression was performed using primers listed in Table 2, and the SYBR® Premix Ex TaqTM (Takara, Dalian, China) on an Applied Biosystems 7500 Fast Real-Time PCR System (Foster City, CA, USA). The total volume of the PCR reaction system was 20 μL. Amplification products were verified by melting curves, agarose gel electrophoresis, and direct sequencing. Results were analyzed by the cycle threshold (CT) method from Fu et al. [22].

**Table 2:** List of gene primer sequences ^a^

Gene name	Prime sequence(5' to 3')		Product size, bp
*GnRH1*	F	GGCTCAACACTGGTCTTATGG	202
	R	TCTTCTGGCTTCTCCTTCG	
*ERR*	F	GTACGGCTCTACTACACTCAGTTATGC	160
	R	CTGCTGGCTGTGGTGATGGATG	
*PGR*	F	GTGTCGCTTGAGGAAGTGCTGTC	116
	R	CGGCTGGCTGCTGAAGTGC	
*LHR*	F	CGTCCTCATAACCAGCCACTACAAG	119
	R	TCTGAGCATCCACCGAAGCAATG	
*FSHR*	F	GTCTCACCTGCTTGCTGATTCTCC	99
	R	CCTTGATCTCCTGGCAGATGAATATCC	
*PPAR-γ*	F	TCCTTCCCGCTGACCAAA	212
	R	TCCTGCACTGCCTCCACA	
*TNF-α*	F	GAGCGTTGACTTGGCTGTC	64
	R	AAGCAACAACCAGCTATGCAC	
*IL-1β*	F	ACTGGGCATCAAGGGCTA	131
	R	GGTAGAAGATGAAGCGGGTC	
*IFN-γ*	F	AGCTGACGGTGGACCTATTATT	259
	R	GGCTTTGCGCTGGATTC	
*IL-4*	F	AGACAAATAACAAAACTGAGC	212
	R	TTGGTGGAAGAAGGTACG	
*NF-κB*	F	GTGTGAAGAAACGGGAACTG	203
	R	GGCACGGTTGTCATAGATGG	
*Caspase1*	F	CGGCCAGCGCCATCTTCATT	347
	R	AGGGAGCTGTCACAGTGCGT	
*IL-12*	F	AAGGTGCAGAAGCAGAGGAC	88
	R	TTGTGTTGCTCTGACTGTTGG	
*TGF-β*	F	TCATCACCAGGACAGCGTTA	109
	R	TGTGATGGAGCCATTCATGT	
*β-actin*	F	GAGAAATTGTGCGTGACATCA	152
	R	CCTGAACCTCTCATTGCCA	

^a^Primers designed using Primer Express software (Sangon Biotech, Shanghai, China)

### Microbial sequencing and analysis

The chyme in the distal region of the ileum was collected at end of 5th week. Sequencing and analysis according to the method described by Zhang et al. [23]. The method was briefly described as that fecal microbial DNA extraction kit (QIAamp Fast DNA Stool Mini Kit, Qiagen Company, Germany) was used to extract microbial DNA from ileal chyme. Nano Drop 2000 (Thermo Scientific, Waltham, MA, USA) was used to determine the concentration of DNA samples, after which 1% agarose gel electrophoresis was used to detect the purity of DNA samples. 16SrDNA gene V3-V4 region universal primers 338 F (5′-ACTCCTACGGGAGGCAGCA-3′) and 806 R (5′-GGACTACHVGGGTWTCTAAT-3′) were used to amplify bacterial DNA, and then the PCR products were purified, quantified and homogenized to form a sequencing library. HiSeq2500 PE250 was used for on-machine sequencing. Sequencing analysis was completed by Beijing Nuohe Zhiyuan Bio-Information Technology Co., Ltd. Qiime software (Qiime2–2019.7, Nature Biotechnology) was used to generate species abundance tables of different taxonomic levels. The alpha diversity and the beta diversity of the samples were analyzed, and then the UPGMA clustering tree was constructed. Subsequently, LEfSe analysis was performed to find biomarkers with statistical differences between the groups based on the LDA value. R software (Version 2.15.3) was used to draw Venn diagram, principal coordinate analysis (P CoA) diagram.

### Statistical analysis

Data were presented as means ± SD and analyzed using one-way ANOVA. Differences among treatment means were determined by Duncan’s post hoc test. Pearson correlation analysis was used to analyze the correlation between ileal bacteria and immune-related indicators at the end of the 5th week. At the end of the trial, the correlation between the indicators related to egg production performance, and the correlation between serum biochemical indicators and egg production performance were also analyzed by Pearson correlation analysis. All statistical analyses were performed by the SPSS 23.0 software (Chicago, IL, USA). Possibility values < 0.05 were taken to indicate statistical significance. Graphpad prism 8.0 software was used to graph the data.

## Results

**Fig. 1:**
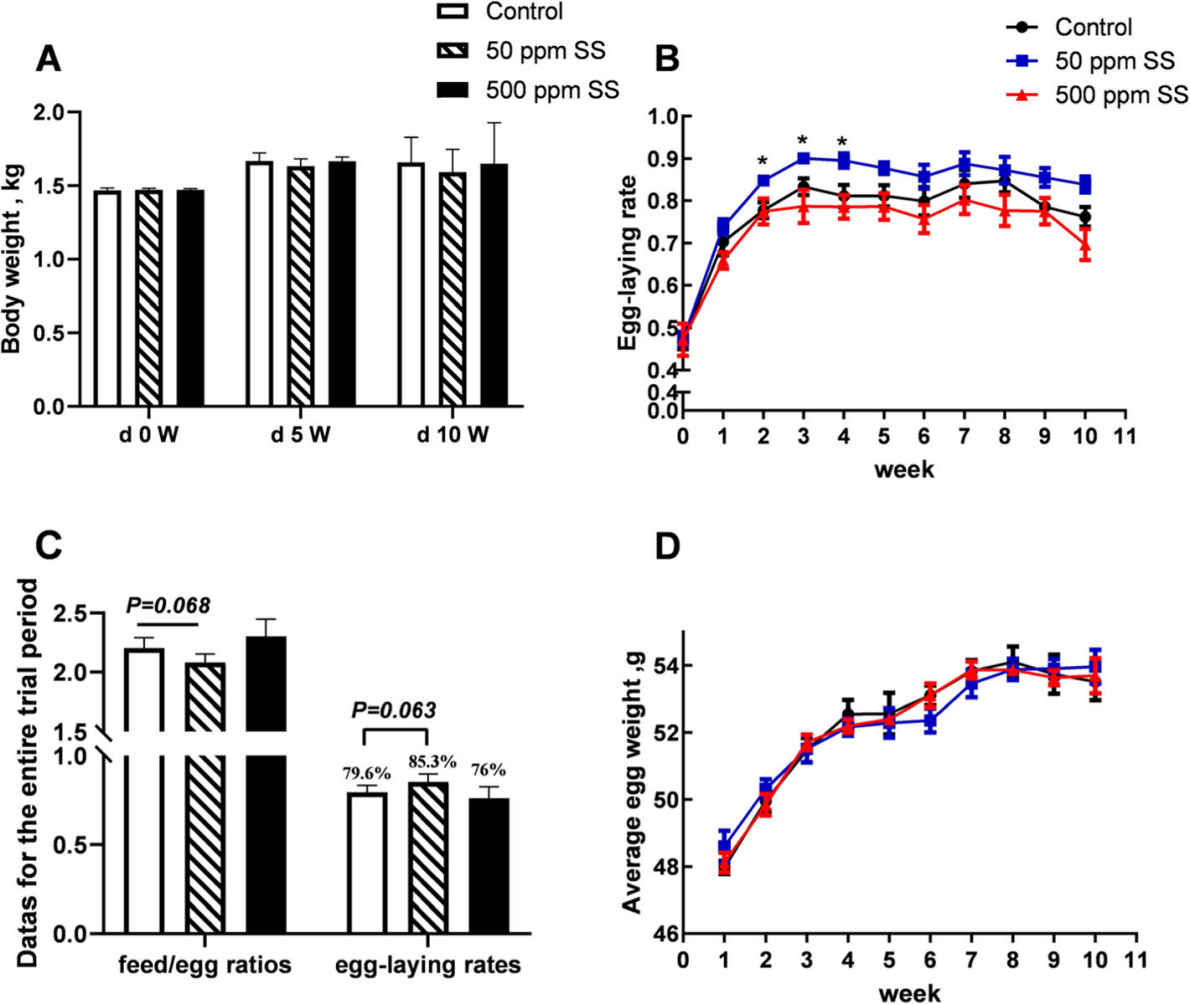
The effects of SS on laying rate and body weight of laying hens. The above **A**, **B**, **C**, and **D** represented the results of body weight, egg production rate, average egg production rate, the ratio of feed to egg (FCR), and average egg weight during the experiment. Among them, * was judged as a significant difference (0.01 < *P* < 0.05), the same below, (*n* = 6)

Compared with the control group, the laying rate and the feed-to-egg ratio tended to be improved in the 50 SS group (0.05 < *P* <  0.1) (Fig. 1C). It should be noticed that egg production rate of laying hens was significantly elevated from 2nd to 4th week (*P* <  0.05) (Fig. 1B). Unexpectedly, during the entire trial period, the egg production rate of 500 SS group was 3.6% lower than that of Control group. With the supplementation of 50 mg/kg SS, the ovarian mass and length index of laying hens at the end of the trial was raised (Tables 3 and 4). The quality of eggshells was improved in 50 and 500 SS group (Table 5).

**Table 3:** Organs weight index

Time	Items	Control	50 ppm SS	500 ppm SS	*P*-value
5th week(27 weeks old, *n* = 6)	Liver, %	2.75 ± 0.44^b^	2.25 ± 0.44^b^	2.12 ± 0.20^a^	0.029
	Spleen, %	0.13 ± 0.02	0.12 ± 0.01	0.12 ± 0.02	0.482
	Ovaries, %	2.69 ± 0.43	2.90 ± 0.51	2.78 ± 0.63	0.791
	Oviducts, %	2.41 ± 1.28	3.43 ± 0.31	2.90 ± 0.97	0.208
10th week(32 weeks old, *n* = 8)	Liver, %	2.40 ± 0.42	2.25 ± 0.22	2.22 ± 0.52	0.625
	Spleen, %	0.09 ± 0.02	0.10 ± 0.01	0.09 ± 0.01	0.176
	Ovaries, %	2.39 ± 0.69^a^	3.20 ± 0.64^b^	2.20 ± 0.67^a^	0.017
	Oviducts, %	2.47 ± 0.44	3.19 ± 0.48	2.99 ± 1.13	0.169

At the end of the 5th and 10th week of the trial, 6–8 birds from each treatment were selected for slaughter to obtain liver, spleen, oviducts, and ovaries. The calculation formula for the weight index of these tissues is as follows: the organ weight, g / live weight of birds, g × 100%. The data in Table 3 different superscript letters indicate significant difference (*P* < 0.05); same superscript letters indicate no difference (*P* > 0.1), the same below

**Table 4:** Results of the oviduct section length

		The length of the oviducts, cm		
Time	Group	Total length	Magnum	Shell gland
5th week(27 weeks old, *n* = 6)	Control	47.98 ± 18.78	22.45 ± 13.25	4.67 ± 1.43
	50 ppm SS	58.33 ± 6.18	33.64 ± 4.40	5.87 ± 1.06
	500 ppm SS	51.73 ± 11.75	27.80 ± 7.36	5.45 ± 1.34
	*P*-value	0.414	0.138	0.291
10th week(32 weeks old, *n* = 8)	Control	54.07 ± 4.16	26.34 ± 6.52	7.32 ± 0.23^ab^
	50 ppm SS	58.40 ± 5.21	32.01 ± 3.58	8.00 ± 0.49^b^
	500 ppm SS	53.88 ± 14.52	29.25 ± 8.14	6.69 ± 1.30^a^
	*P*-value	0.55	0.228	0.015

At the end of the 5th and 10th week of the trial, 6–8 birds from each treatment were selected for slaughter to obtain oviducts, and the total length of oviducts, magnum, and shell gland were measured. The data in Table 4 different superscript letters indicate significant difference (*P* < 0.05); same superscript letters indicate no difference (*P* > 0.1), the same below

**Table 5:** Egg quality results (*n* = 11)

Time, week	Group	Eggshell strength, kg/cm^2^	Albumen hight, mm	Haugh unit	Egg yolk color	Eggshell thickness, mm
1st-3rd (23–25 weeks old)	Control	4.27 ± 0.28^a^	8.93 ± 1.00	94.61 ± 5.82	7.85 ± 0.42	0.351 ± 0.015^a^
	50 ppm SS	4.50 ± 0.28^ab^	9.05 ± 1.71	93.51 ± 8.61	7.73 ± 0.36	0.349 ± 0.016^a^
	500 ppm SS	4.73 ± 0.33^b^	9.16 ± 1.45	97.37 ± 6.42	7.82 ± 0.34	0.364 ± 0.007^b^
	*P*-value	0.004	0.931	0.428	0.744	0.026
4th–6th (26–28 weeks old)	Control	4.53 ± 0.18	8.21 ± 1.48^ab^	90.85 ± 8.29	7.48 ± 0.28	0.366 ± 0.010^a^
	50 ppm SS	4.73 ± 0.53	8.72 ± 0.49^b^	92.59 ± 3.98	7.67 ± 0.37	0.375 ± 0.009^b^
	500 ppm SS	4.52 ± 0.10	7.38 ± 0.89^a^	88.49 ± 6.50	7.73 ± 0.25	0.365 ± 0.008^a^
	*P*-value	0.263	0.017	0.345	0.161	0.03
7th–9th (29–31 weeks old)	Control	4.47 ± 0.47^a^	8.85 ± 0.71	94.31 ± 5.57	7.21 ± 0.40	0.363 ± 0.015^a^
	50 ppm SS	4.94 ± 0.48^b^	8.90 ± 0.73	94.37 ± 4.02	7.31 ± 0.27	0.378 ± 0.013^b^
	500 ppm SS	4.24 ± 0.42^a^	9.35 ± 1.14	97.20 ± 4.31	7.09 ± 0.37	0.359 ± 0.009^a^
	*P*-value	0.004	0.362	0.271	0.346	0.003

During the trial, a repeat group was randomly selected from each treatment every 3 weeks, and all the eggs in this repeat group were collected for egg quality determination. The data in Table 5 different superscript letters indicate significant difference (*P* < 0.05); same superscript letters indicate no difference (*P* > 0.1), the same below

**Fig. 2:**
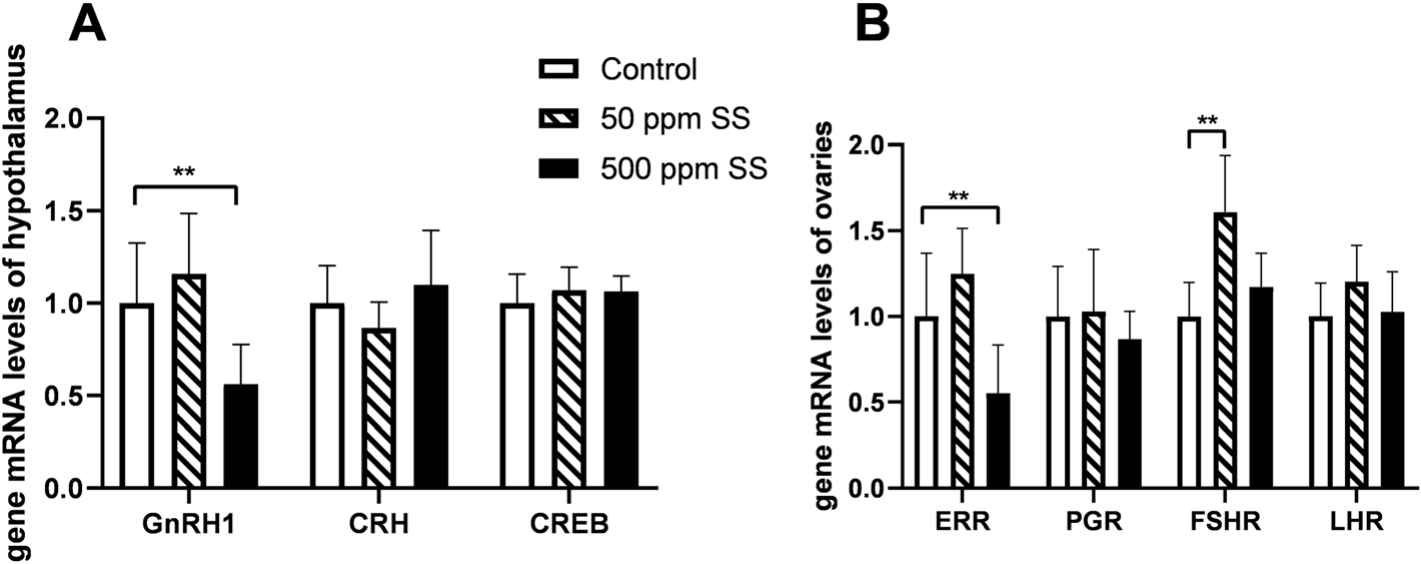
The effect of SS on the gene mRNA levels about hypothalamus and ovary. The above **A** and **B** represented the results of gene mRNA levels about hypothalamus and ovary at the end of the trial. Among them, ** represents an extremely significant difference (*P* < 0.01), the same below (*n* = 8)

With 50 mg/kg SS added, the serum FSH level was elevated at the end of the trial (*P* <  0.05) (Table 6). The contents of E2, P and LH in the serum were significantly elevated at the end of 5th week in the 500 SS group (*P* <  0.05). However, the levels of E2 and LH in the serum were reduced numerically at the end of the trial. The mRNA levels of follicle stimulating hormone receptor (*FSHR)* in ovarian was up-regulated with 50 mg/kg SS supplementation (*P* < 0.01) (Fig. 2B). Interestingly, the mRNA levels of genes such as gonadotropin releasing hormone 1 (*GnRH1*) in hypothalamus and the estrogen related receptor (*ERR*) were down-regulated in the 500 SS group (*P* < 0.01) (Fig. 2A, B). In order to further clarify, we conducted a correlation analysis of indicators related to egg production performance at the end of the trial. Results showed that some indicators such as the total oviducts length, shell gland length, the mRNA levels of *ERR* and *FSHR* in ovarian, and the mRNA level of *GnRH1* in hypothalamus were highly positively correlated with egg production rate (*P* < 0.05) (Table 7).

**Table 6:** Serum E2, P, LH, FSH levels

Time	Group	E2, pg/mL	P, ng/mL	LH, mIU/mL	FSH, mIU/mL
5th week(27 weeks old, *n* = 6)	Control	682.26 ± 160.43^a^	0.25 ± 0.04^a^	3.54 ± 0.60^a^	2.48 ± 0.55
	50 ppm SS	782.42 ± 166.81^ab^	0.25 ± 0.07^a^	4.63 ± 0.95^a^	2.85 ± 0.53
	500 ppm SS	928.54 ± 133.90^b^	0.41 ± 0.10^b^	8.76 ± 1.68^b^	3.17 ± 0.73
	*P*-value	0.026	0.001	< 0.001	0.132
10th week(32 weeks old, *n* = 8)	Control	646.17 ± 128.77^ab^	0.23 ± 0.08^a^	5.11 ± 1.74	2.35 ± 0.62^a^
	50 ppm SS	701.59 ± 98.47^b^	0.37 ± 0.08^b^	5.76 ± 1.16	3.11 ± 0.81^b^
	500 ppm SS	537.52 ± 115.01^a^	0.30 ± 0.08^ab^	4.38 ± 1.43	2.13 ± 0.49^a^
	*P*-value	0.029	0.007	0.197	0.017

At the end of the 5th and 10th week of the trial, 6–8 birds from each treatment were selected to obtain wing vein blood and harvest serum. The levels of E2, P, LH, and FSH in the serum were measured. The data in Table 6 different superscript letters indicate significant difference (*P* < 0.05); same superscript letters indicate no difference (*P* > 0.1), the same below

**Table 7:** Correlation analysis of related indexes about egg production performance at the end of the trial (* represents 0.01<*P*<0.05, ** represents *P*<0.01)

	E2	P	LH	FSH	Egg-laying rate	Ovaries weight %	Oviducts weight %	Total oviducts length	Magnum length	Shell gland length	*GnRH1*	*ERR*	*PGR*	*LHR*	*FSHR*
E2	1														
P	0.244	1													
LH	0.005	−0.120	1												
FSH	0.201	0.176	0.447*	1											
Egg-laying rate	0.153	0.251	0.170	−0.082	1										
Ovaries weight %	0.365	0.193	0.194	0.358	0.061	1									
oviducts weight %	−0.003	0.048	−0.093	−0.040	0.167	0.198	1								
Total oviducts length	−0.110	−0.101	0.096	0.009	0.488*	0.158	0.712**	1							
Magnum length	−0.011	0.100	0.016	0.026	0.302	0.067	0.642**	0.757**	1						
Shell gland length	0.330	0.111	0.182	0.283	0.786**	0.424*	0.389	0.625**	0.379	1					
*GnRH1*	0.438*	0.051	0.153	0.212	0.482*	0.238	0.063	0.203	0.214	0.312	1				
*ERR*	0.283	0.095	0.166	0.171	0.672**	0.358	−0.046	0.316	0.303	0.454*	0.707**	1			
*PGR*	0.235	0.020	−0.094	0.069	0.035	0.026	−0.185	−0.045	0.069	−0.061	0.474*	0.538**	1		
*LHR*	0.332	0.217	−0.009	−0.002	0.316	0.509*	0.256	0.366	0.177	0.336	0.114	0.202	0.107	1	
*FSHR*	0.274	0.518**	0.186	0.311	0.524*	0.269	0.213	0.306	0.284	0.422*	0.305	0.451*	0.419*	0.320	1

**Fig. 3:**
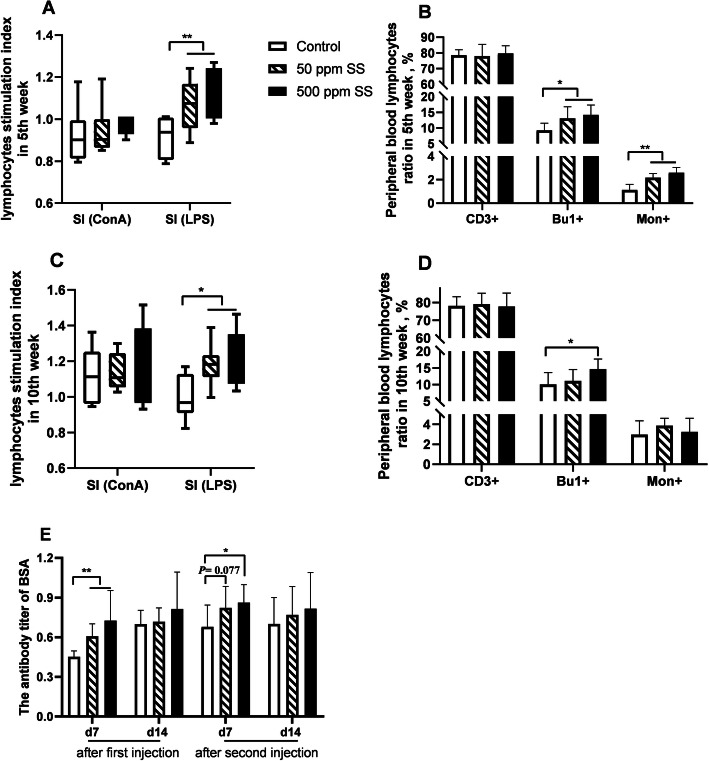
The effects of SS on peripheral blood lymphocyte ratio, stimulation index, and the antibody titer of BSA. Peripheral blood lymphocyte stimulation index and lymphocyte ratio at the end of 5th week were showed in **A** and **B**, (*n* = 6). **C** and **D** showed the peripheral blood lymphocyte stimulation index and lymphocyte ratio at the end of 10^th^ week, (*n* = 8). The results of BSA antibody titer were showed in **E**, (*n* = 8). Among them, ** represents an extremely significant difference (*P* < 0.01), * was judged as a significant difference (0.01 < *P* < 0.05), the same below. Our analysis steps for flow cytometry results were as follows. At first, we use the CD45 ring gate to eliminate the interference of red blood cells. In the gate of CD45^+^, T lymphocytes were labeled with CD3^+^ and their ratios were obtained. B lymphocytes and monocytes were labeled with Bu1^+^ and Mon^+^, and their ratios were obtained. The detailed flow analysis density map could be tracked in supplementary Figs. 1 and 2

In addition to egg laying performance, we also explored the impact of SS on the immune function and the structure of intestinal flora. Results showed that at the end of 5th and 10th week, the peripheral blood LPS stimulation index and the proportion of B lymphocytes were significantly increased in the 50 and 500 SS group (Fig. 3A, B, C, D). The antibody titer after 7 days of first immunization with BSA was elevated with 50 and 500 mg/kg SS addition. In addition, the antibody titer 7 days after the second immunization with BSA was raised with 500 mg/kg SS supplementation (*P* < 0.05) (Fig. 3E). The contents of serum IgG and IgM were heightened in the 50 and 500 SS group at the end of trial (*P* < 0.05) (Table 8). The results of serum cytokines levels told us that the levels of IL-4 and IFN-γ were elevated in the 50 and 500 SS group at the end of the trial (*P* < 0.05) (Table 9), however, the ratio of IFN-γ to IL-4 was heightened with 500 mg/kg SS supplementation (*P* < 0.05).

**Table 8:** Serum immunoglobulin levels in 5th and 10th week

	Item	Control	50 ppm SS	500 ppm SS	*P*-value
5th week(27 weeks old, *n* = 6)	IgG, ng/mL	1156.99 ± 52.49	1149.89 ± 35.00	1142.72 ± 46.55	0.863
	IgA, ng/mL	296.73 ± 14.41	306.59 ± 4.67	309.71 ± 5.80	0.072
	IgM, ng/mL	86.33 ± 14.59	100.28 ± 22.81	98.09 ± 15.22	0.374
10th week(32 weeks old, *n* = 8)	IgG, ng/mL	1109.62 ± 91.10^a^	1262.94 ± 92.65^b^	1224.45 ± 126.08^b^	0.021
	IgA, ng/mL	294.04 ± 12.71	303.03 ± 46.22	307.40 ± 32.11	0.719
	IgM, ng/mL	70.11 ± 10.80^a^	94.08 ± 11.95^b^	85.87 ± 16.77^b^	0.006

At the end of the 5th and 10th week of the trial, 6–8 birds from each treatment were selected to obtain wing vein blood and harvest serum. The levels of IgG, IgA, and IgM in the serum were measured. The data in Table 8 different superscript letters indicate significant difference (*P* < 0.05); same superscript letters indicate no difference (*P* > 0.1), the same below

**Table 9:** Serum cytokine levels in 5th and 10th week

	Item	Control	50 ppm SS	500 ppm SS	*P*-value
5th week(27 weeks old, *n* = 6)	IL-2, pg/mL	72.05 ± 4.25	66.95 ± 4.88	70.11 ± 4.92	0.198
	IL-6, pg/mL	83.24 ± 9.76	88.42 ± 14.52	91.17 ± 18.16	0.640
	IL-4, pg/mL	20.08 ± 2.83^a^	23.45 ± 4.47^ab^	29.87 ± 8.57^b^	0.032
	IFN-γ, pg/mL	62.47 ± 13.16	67.01 ± 10.64	82.20 ± 21.04	0.104
	IFN-γ/IL-4	3.10 ± 0.42	2.93 ± 0.60	2.78 ± 0.16	0.450
10th week(32 weeks old, *n* = 8)	IL-2, pg/mL	70.98 ± 5.14	73.56 ± 8.82	74.66 ± 9.22	0.643
	IL-6, pg/mL	101.90 ± 18.43	111.58 ± 25.05	120.06 ± 22.58	0.283
	IL-4, pg/mL	21.21 ± 6.61^a^	28.99 ± 5.92^b^	26.02 ± 4.35^ab^	0.039
	IFN-γ, pg/mL	55.04 ± 14.61^a^	66.13 ± 14.34^ab^	78.17 ± 12.35^b^	0.011
	IFN-γ/IL-4	2.63 ± 0.24^ab^	2.33 ± 0.52^a^	3.04 ± 0.48^b^	0.012

At the end of the 5th and 10th week of the trial, 6–8 birds from each treatment were selected to obtain wing vein blood and harvest serum. The levels of some cytokines in the serum were measured. The data in Table 9 different superscript letters indicate significant difference (*P* < 0.05); same superscript letters indicate no difference (*P* > 0.1), the same below

**Fig. 4:**
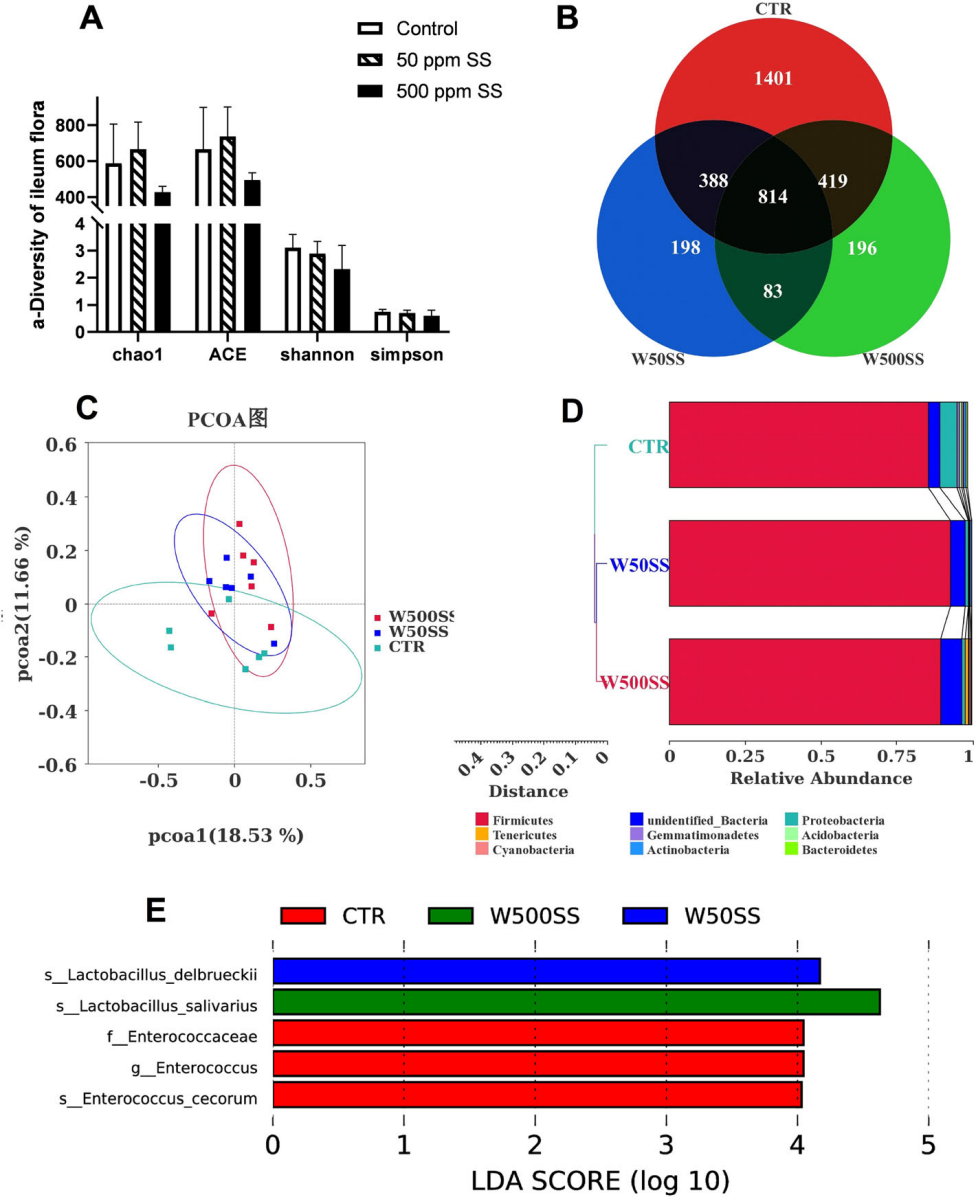
The effect of SS on the microbial structure of the ileum of laying hens at the end of the 5th week (27 week old). **A**, **B**, **C**, **D**, and **E** represented the α-diversity of the flora, the venn diagram of different species, the β-diversity (PcoA), the cluster structure of the flora, and the results of the differential flora analyzed by LEFSe. Among them, WCTR = Control, WC50SS = 50 ppm SS, WC500SS = 500 ppm SS (*n* = 6)

**Fig. 5:**
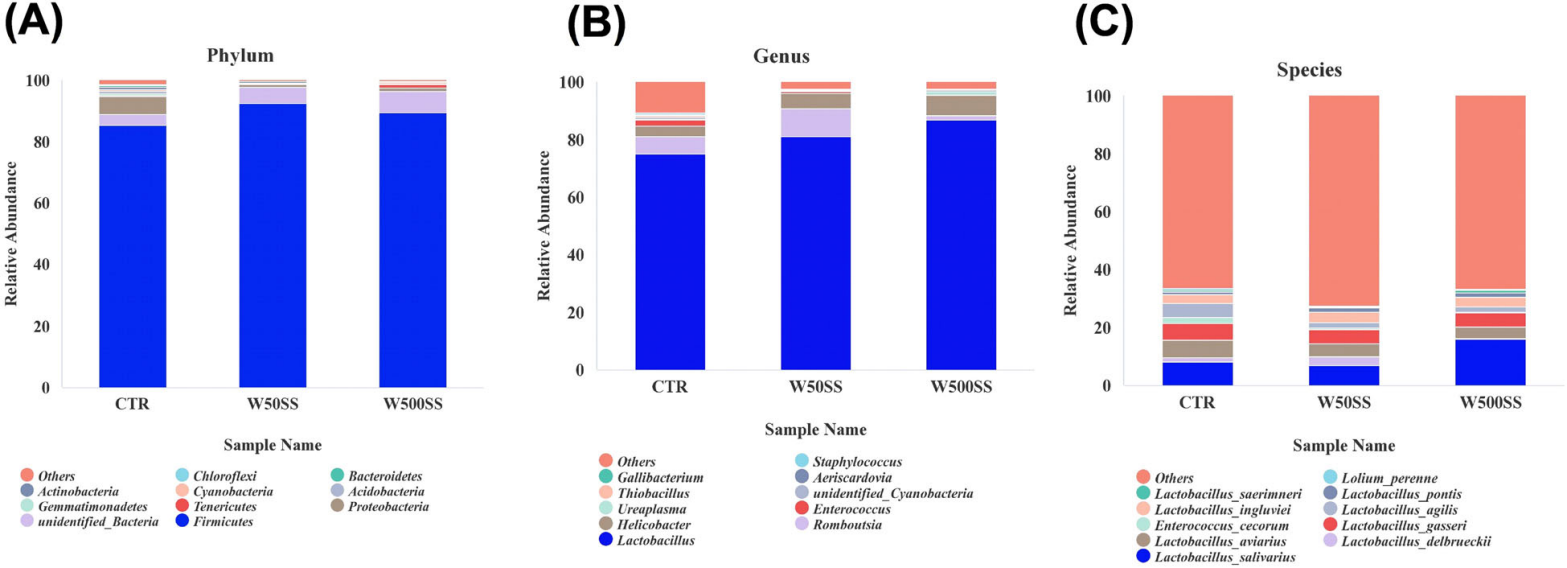
The effect of SS on the relative abundance of bacteria at different classification levels at the end of 5th week (27th week). The above figure 5A, 5B, and 5C respectively represent the relative abundance results of ileal flora at the phylum, genus and species level. Among them, CTR = Control, W50SS = 50 ppm SS, W500SS = 500 ppm SS (*n* = 6)

**Fig. 6:**
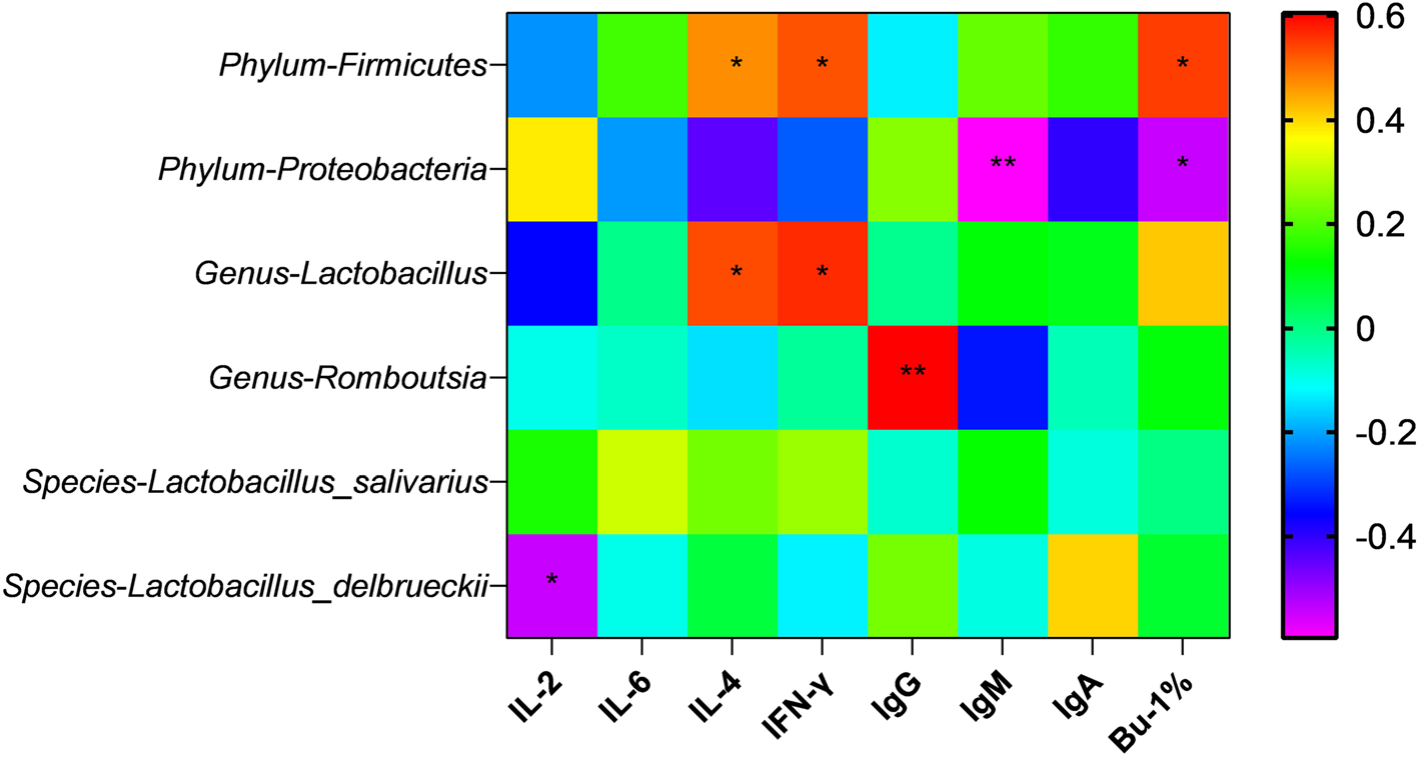
The correlation analysis results between intestinal bacteria and immune-related indicators at the end of 5th week (27 week old). The results of the correlation analysis between the immune-related indicators and the bacteria with differences at the end of 5th week were showed in Fig. 6. Among them, * represents a significant difference (0.01 < *P* < 0.05), and ** represents an extremely significant difference (*P* < 0.01)

LEfSe analysis further showed that *Lactobacillus delbrueckii* was the dominant flora in the 50 SS group, and *Lactobacillus_salivarius* was regarded as the dominant flora in the 500 SS group (Fig. 4E). Compared with the control group, the relative abundance of Lactobacillus and *Romboutsia* (Fig. 5B) in the ileum chyme were elevated, and the relative abundance of *Proteobacteria* (Fig. 5A) was decreased in the 50 SS group. Clearly, the relative abundance of *Lactobacillus salivarius* was increased, and the relative abundance of Proteobacteria was reduced with 500 mg/kg SS (Fig. 5A, 5C). The results of the correlation analysis between the bacteria and immune indicators at the end of 5th week showed that Firmicutes and *Lactobacillus* were positively correlated with the level of IL-4, IFN-γ and the ratio of B cell in the blood (*P* < 0.05). Proteobacteria was significantly negatively correlated with the contents of IgM and the ratio of B cell in the blood (*P* < 0.05). Additionally, *Lactobacillus delbrueckii* was negatively correlated with the level of serum IL-2. *Romboutsia* was positively correlated with the level of IgG in serum (*P* < 0.05) (Fig. 6).

**Fig. 7:**
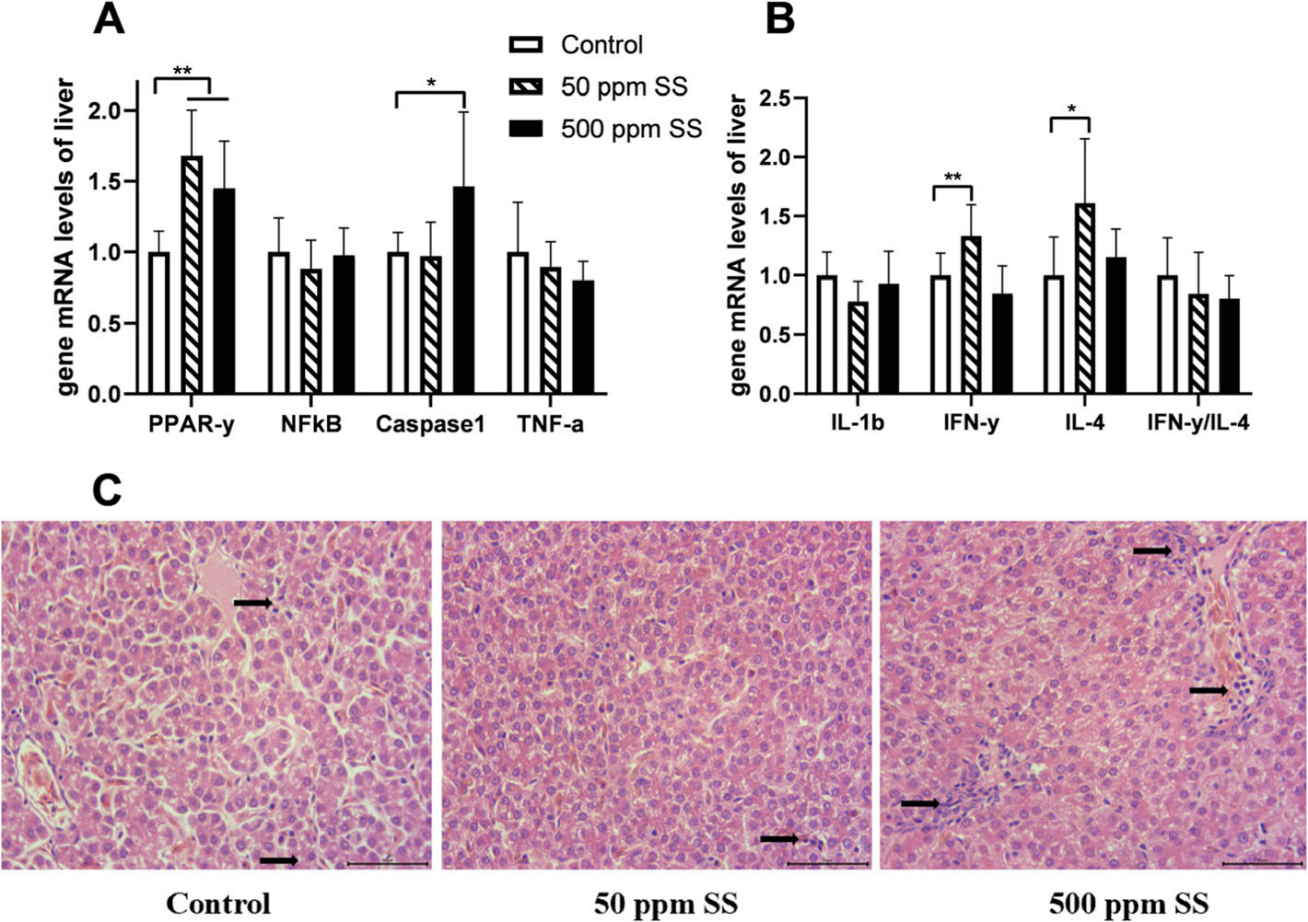
The effect of SS on the gene mRNA levels and morphology of liver. The above **A** and **B** represented the results of gene mRNA levels about liver at the end of the trial. The morphological observation results about the liver at the end of the trial were showed in **C** above. The arrow points to the infiltration of immune cells. Among them, ** represents an extremely significant difference (*P* < 0.01), * was judged as a significant difference (0.01 < *P* < 0.05), the same below (*n* = 8)

**Fig. 8:**
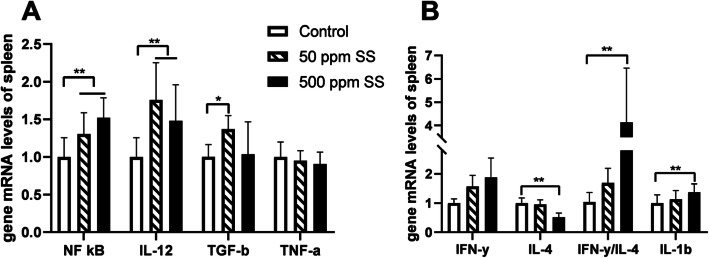
The effect of SS on the gene mRNA levels of spleen. The results of gene mRNA levels about spleen at the end of the trial were showed in the above **A** and **B**. Among them, ** represents an extremely significant difference (*P* < 0.01), * was judged as a significant difference (0.01 < *P* < 0.05), the same below (*n* = 8)

**Fig. 9:**
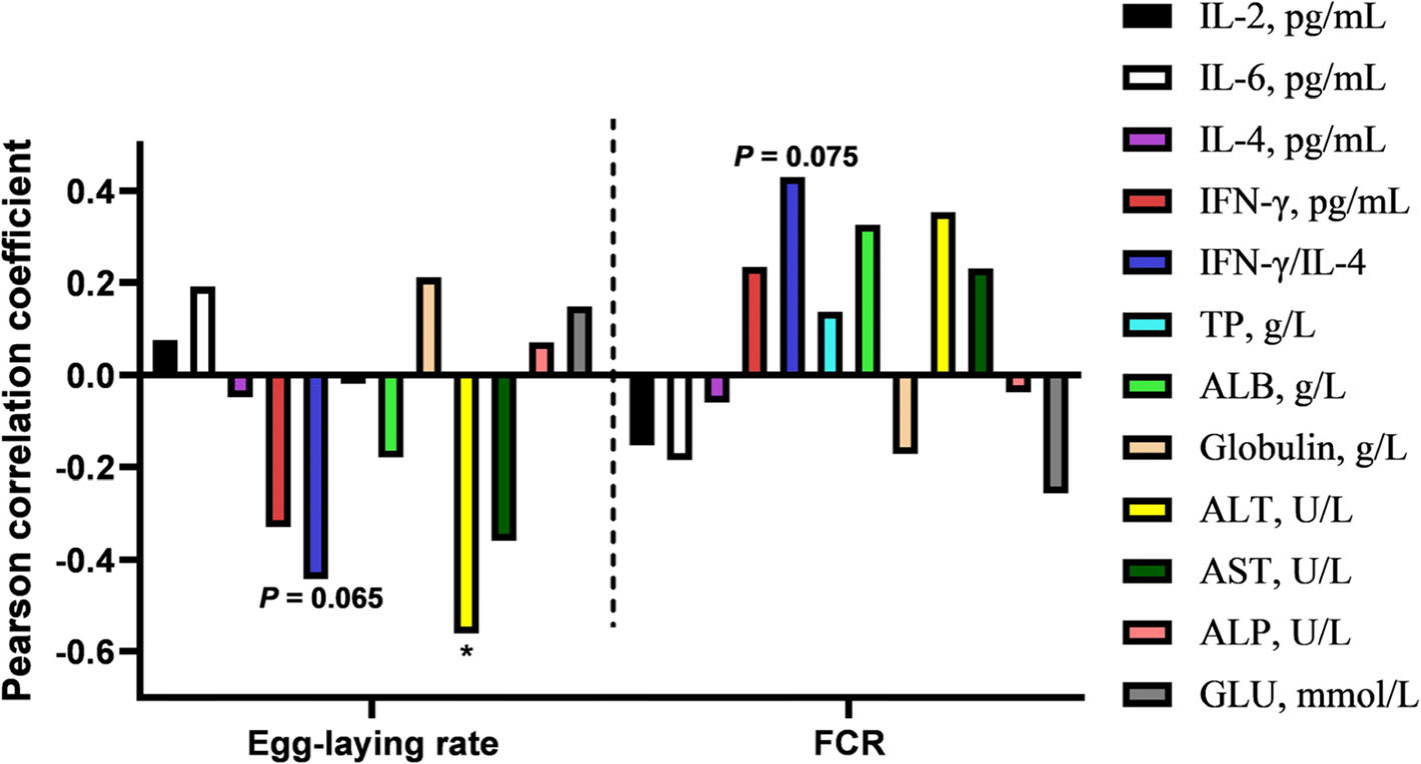
Correlation analysis between serum biochemical indicators and egg production performance at the end of the trial. The results of correlation analysis between serum biochemical indicators and egg production performance at the end of the trial were showed in Fig. 9. Among them, * represents a significant difference (0.01 < *P* < 0.05)

The results of mRNA levels in liver and spleen showed that *IL-4* and *IFN-γ* in liver, nuclear transcription factor kappa B (*NF-κB),* interleukin-12 (*IL-12),* transforming growth factor *(TGF-β*) and *IFN-γ* in spleen were up-regulated at the end of the trial in the 50 SS group (*P* < 0.05) (Figs. 7B, 8A, B). With 500 mg/kg SS supplemented, the gene mRNA levels of cysteinyl aspartate specific proteinase 1 (*Caspase-1*) in liver, *NF-κB, IL-12,* Interleukin-1β (*IL-1β*) and the ratio of *IFN-γ* to *IL-4* about spleen were up-regulated (*P* < 0.05). We also found that the levels of serum alanine aminotransferase (ALT) and alkaline phosphatase (ALP) were elevated in the 500 SS group (Table 10). Moreover, we observed from the liver tissue morphology that there was more immune cell infiltration of liver in the 500 SS group (Fig. 7C). Interestingly, the level of ALT and the ratio of IFN-γ to IL-4 in the serum were negatively correlated with the egg-laying rate (Fig. 9).

**Table 10:** The results of serum biochemical index at the end of the trial (*n* = 8)

Item	Control	50 ppm SS	500 ppm SS	*P*-value
TP, g/L	32.63 ± 1.27	32.46 ± 0.78	32.47 ± 0.76	0.927
ALB, g/L	12.57 ± 0.98	11.88 ± 0.88	12.25 ± 1.01	0.377
Globulin, g/L	19.70 ± 0.70^a^	20.58 ± 0.74^b^	20.22 ± 0.91^ab^	0.106
ALT, U/L	14.02 ± 1.55^a^	13.44 ± 1.54^a^	15.56 ± 1.07^b^	0.019
AST, U/L	111.80 ± 8.07	111.70 ± 8.12	117.69 ± 14.38	0.447
ALP, U/L	313.08 ± 21.85^a^	369.94 ± 13.68^b^	379.88 ± 28.54^b^	< 0.001
GLU, mmol/L	12.15 ± 0.15	12.23 ± 0.33	12.19 ± 0.50	0.904

At the end of the trial, 8 birds from each treatment were selected to obtain wing vein blood and harvest serum. The levels of serum biochemical index were measured. The data in Table 10 different superscript letters indicate significant difference (*P* < 0.05); same superscript letters indicate no difference (*P* > 0.1), the same below

## Discussion

In the present study, the egg production rate and FCR were improved with 50 mg/kg SS supplementation. It has been suggested that ginsenosides promotes the proliferation of ovarian cells in chicken [14–16], We could probably argue that SS might improve the development of the reproductive tract to regulate hormone secretion, thereby increasing the egg production rate. The physiological factors, especially endocrine factors regulate egg production via gonadotropin releasing hormone (GnRH), estradiol (E2), prolactin (PRL), follicle stimulating hormone (FSH) and luteinizing hormone (LH). Importantly, the secretion of these hormones is controlled via the “hypothalamus-pituitary-ovarian” axis [24]. In the present study, the contents of E2 and FSH in the serum at 10th week were elevated with 50 mg/kg SS addition. As a major estrogen produced in the ovaries, estradiol could promote the development of the reproductive system to improve the reproductive performance of the body. While FSH promotes the production of sperm in poultry or other vertebrates and stimulates the maturation of ovarian follicles [25, 26]. Furthermore, a study showed that the ovarian FSH receptor (FSHR) stimulated the growth of follicles and regulates ovarian development via mediating estrogen synthesis [27]. In the present study, the length of shell gland and the mRNA level of *FSHR* in the ovarian tissue were elevated with 50 mg/kg SS addition. We also found that the length of shell gland, the mRNA levels of *ERR* and *FSHR* in ovarian were highly positively correlated with egg production rate. It seems plausible that dietary 50 mg/kg SS might improve egg production rate via improving the development of ovary and ovulation and stimulating the secretion of E2 and FSH.

This study showed that dietary 500 mg/kg SS significantly increased the level of E2, P, LH in the serum at the end of 5th week. Paradoxically, the content of E2 in the serum tended to decrease at the end of the trial. It has been reported that progesterone stimulates the pituitary gland to secrete FSH and LH to promote follicular maturation and ovulation. The increase in progesterone levels followed by higher levels of FSH and LH could be considered as an important indicator of ovarian function decline [28]. Likewise, E2 regulates ovarian activity through the feedback function of hypothalamus and pituitary. Decreased serum E2 level may cause ovarian dysfunction, premature ovarian failure and down’s Syndrome [29, 30]. A possible explanation could be that the high-dose SS in the early stage of the experiment excessively stimulated the laying hens to secrete P, E2 and LH, which caused the ovarian function to decline. At the same time, the high-dose estrogen negative feedback regulated the “hypothalamus-pituitary-ovarian” axis. Thus, ovarian estrogen secretion was reduced. In the present study, the results of association analysis showed that the mRNA levels of *ERR* in ovarian, and *GnRH1* in hypothalamus were highly positively correlated with egg production rate. we also found the mRNA levels of hypothalamus *GnRH1* and ovary *ERR* were significantly down-regulated with 500 mg/kg SS supplemented at the end of trial. Although we did not observe a significant difference in egg production rates between control and the 50 SS groups, a 3.6% reduction of egg production in 500 SS group is unacceptable for producers.

We observed a positive impact of SS supplementation on egg shell characteristic during the trial period. Past finding showed that SS Bb promotes the absorption of zinc via increasing the expression of zinc transporter protein 4 (Zip4) protein in cells [31]. Also, SS promote the absorption and utilization of calcium via promoting the absorption and utilization of zinc [32]. This could explain why a positive impact of SS was observed in our study, as well.

Serum globulin level was used as an indicator to assess the innate immunity of animals [33]. In the present study, the serum globulin level was significantly elevated in the 50 SS group at the end of the trial. Additionally, the proliferation and differentiation of peripheral blood lymphocytes directly determine immune function of the body. It is generally accepted that ConA promotes the proliferation of T cells, while LPS could directly stimulate the proliferation and differentiation of mature B cells [20]. It is commonly known that B cells are mainly involved in mediating fluid immune function, some researchers regarded the serum levels of total IgG, IgM and IgA as the basis for assessing the humoral immunity of poultry [34]. In our study, we also found that the stimulation index of LPS to peripheral blood lymphocytes and proportion of B lymphocytes were significantly raised with 50 and 500 mg/kg SS supplemented. It is tempting to believe that SS might improve the immune function by regulating the humoral immune response. Interestingly, we also found that the levels of serum IgG and IgM were raised with 50 and 500 mg/kg SS addition. The serum levels of BSA antibody titer induced by intramuscular injection of BSA are regarded as the basis for assessing the humoral immunity of poultry [34]. In the present study, the antibody titer after 7 days of immunization with BSA was elevated with 50 and 500 mg/kg SS addition. These evidences suggested that dietary 50 and 500 mg/kg SS might improve the immune function via regulating the humoral immune response of laying hens. It was basically consistent with the results reported by Naveed et al. [10].

Cytokines play an important role in regulating the immune function of the body. The coordinated expression of these pro-inflammatory and anti-inflammatory cytokines maintains the body’s immune homeostasis [35, 36]. The typical representatives of pro-inflammatory and anti-inflammatory cytokines are IFN-γ and IL-4, respectively. The ratio of IFN-γ to IL-4 was used to reflect the immune homeostasis of body [37]. In the present study, the level of serum IL-4 was elevated in the 50 and 500 SS group at the end of 5th and 10th week. Study suggested that the expression of IL-4 stimulates the proliferation of B cells [35]. This might further prove that SS improved the humoral immune function of laying hens. To explain the observed activity, we might consider that the complex polysaccharide molecular structure of SS stimulated immune cells such as activated T cells to secrete cytokines, and the immune function of body was improved. Further research is needed to clearly understand this mechanism.

It is well established that the structure of the intestinal flora is closely related to the host’s immune function. In the intestinal flora, bacteria of the genus *Lactobacillus* tend to metabolize to produce SCFAs and functional oligosaccharides, etc. These beneficial metabolites improve host immune function. However, some harmful bacteria, for instance pathogenic *Escherichia coli*, *Campylobacter jejuni* and *Helicobacter pylori* in the phylum Proteus metabolize and produce some endotoxins which affect the health of the host [38, 39]. We found that the relative abundance of *Lactobacillus* in the ileal chyme was elevated, and the relative abundance of Proteobacteria was decreased in the 50 and 500 SS group. On the basis of these results, we concluded that an appropriate level of SS could improve the intestinal microflora of laying hens.

*Romboutsia* is a group of bacteria that ferment multiple carbohydrates and metabolize to produce SCFAs, oligosaccharides and other prebiotics. Studies found that *Romboutsia* was closely related to obesity, Crohn’s disease and diabetes [40–42]. *Romboutsia* was also supposed to be the key role in regulating immune function [43]. Studies suggested that with the *Lactobacillus delbrueckii* supplemented, the immune function of Yellow River carp and mice were improved via elevating the level of serum lysozyme and IgM [44–46]. In the present study, the relative abundance of *Romboutsia* and *Lactobacillus delbrueckii* in ileal chyme were significantly heightened with 50 mg/kg SS supplemented. Studies also found that with *Lactobacillus salivarius* added to the diet of broiler, the antibody titer of the serum infectious bursal disease virus (IBDV) vaccine was increased, and the level of serum lysozyme was also elevated [35, 47]. In our study, the relative abundance of *Lactobacillus salivarius* in the ileal chyme was heightened in the 500 SS group. We also found that Firmicutes and *Lactobacillus* were positively correlated with the levels of IL-4, IFN-γ, and the ratio of B cell in the blood. Proteobacteria was significantly negatively correlated with the contents of IgM and the ratio of B cell. Additionally, *Romboutsia* were positively correlated with the content of IgG in serum. It illustrated us that dietary 50 and 500 mg/kg SS might improve the immune function via improving intestinal flora during the first 5 weeks of the trial. It should be mentioned that *Romboutsia*, *Lactobacillus delbrueckii, and Lactobacillus salivarius* might participate in the metabolic process of SS in the intestine, and then produce some biologically active substances to regulate immune function. Further research is needed to clearly understand this mechanism.

NF-κB is the central regulator of cellular stress in all cell types of the body, it participates in the regulation of innate and adaptive immunity, as well as cell turnover [48]. Under non-pathological conditions, the body’s immune system is activated by NF-κB to resist the stimulation of pathogens. However, once NF-κB is excessively activated, the overexpression of pro-inflammatory cytokines such as IL-1β and TNF-α lead body to an inflammatory response [49]. At the end of the trial, we found the mRNA levels of genes such as *NF-κB, IL-12, TGF-β,* and *IFN-γ* in spleen were up-regulated in the 50 SS group. Additionally, the levels of IFN-γ and IL-4 in the serum were also heightened. We also found that the ratio of IFN-γ and IL-4 was not different from that of the control group, it indicated that the body was in immune homeostasis [37]. Thus, we hold that the increase of those cytokines makes a hen more responsive to challenges. It lighted us that dietary 50 mg/kg SS for 10 weeks could improve the immune function of laying hens. Unexpectedly, one unanticipated finding was that the mRNA levels of *NF-κB, IL-12, IFN-γ, IL-1β* and the ratio of *IFN-γ* to *IL-4* in spleen were up-regulated as well as the level of serum IFN-γ in the 500 SS group. In addition, the transcription level of spleen *IL-4* was down-regulated, and the ratio of IFN-γ to IL-4 in serum tended to be elevated. We despondently believed that dietary 500 mg/kg SS for 10 weeks could break immune homeostasis. It might have a negative effect on laying hens. To gain more insight, the following test was studied.

Caspase 1 is involved in the proteolytic activation of IL-1β family cytokines. When the body is in a state of stress, Caspase 1 will be activated [50]. The levels of ALT and AST in the blood are often used as indicators to evaluate liver function [51]. In the present study, the mRNA level of *Caspase1* in liver, and the contents of ALT and ALP in serum were significantly raised in the 500 SS group. We also found more inflammatory cell infiltration in the liver of this group. Although we did not find obvious signs of inflammation, dietary 500 mg/kg SS did have a negative effect on the liver of laying hens. Additionally, the results of the correlation analysis showed that the level of ALT and the ratio of IFN-γ to IL-4 in the serum were negatively correlated with the egg-laying rate. It illuminated us that the long-term load of organs would inevitably have a negative impact on production performance. As we found in our study, the egg production rate of 500 SS group was 3.6% lower than that of Control group. To explain the observed activity, we might consider that a high dose of SS might stimulate the immune system for a period of time to improve the immune function. However, with prolonged immune stimulation response, the nutritional base of body will be consumed, and the organs would be overloaded. As a result, the production performance would be negatively affected.

## Conclusion

Dietary 50 mg/kg SS improved the egg production performance via stimulating ovaries development, increasing ovarian *FSHR* transcription level and serum estrogen level. The intestinal microflora was regulated, and the immune function of laying hens also was improved with 50 mg/kg SS supplementation. The long-term supplementation of 500 mg/kg SS exerted a negative impact on the laying performance and physiological functions of the liver of laying hens.

## Supplementary Information

**Additional file 1: Supplementary materials.** The flow cytometry density map of peripheral blood at the end of 5th week (27 weeks old, *n* = 6). Supplementary materials: the flow cytometry density map of peripheral blood at the end of 10th week (32 weeks old, *n* = 8).

## Data Availability

The datasets produced and/or analyzed during the current study are available from the corresponding author on reasonable request.

## Abbreviations

ALB: Albumin
ALT: Alanine aminotransferase
ALP: Alkaline phosphatase
AST: Aspartate aminotransferase
BSA: Bovine serum albumin
Caspase1: Cysteinyl aspartate specific proteinase 1
Con A: Concanavalin A
DNFB: 2,4-dinitro fluoro benzene
E2: Estradiol
ERR: Estrogen related receptor
FSH: Follicle stimulating hormone
FSHR: Follicle stimulating hormone receptor
GLU: Glucose
GnRH1: Gonadotropin releasing hormone 1
IL-1β: Interleukin 1β
IL-4: Interleukin 4
IL-12: Interleukin 12
IFN-γ: Interferon γ
LH: Luteinizing hormone
LHR: Luteinizing hormone receptor
LPS: Lipopolysaccharide
NF-κB: Nuclear transcription factor kappa B
P4: Progesterone
PGR: Progesterone receptor
PPAR-γ: Peroxisome proliferator activated receptor γ
TNF-α: Tumor necrosis factor α
TGF-β: Transforming growth factor β
