# Supplementary material for: Soya saponin improves egg-laying performance and immune function of laying hens
Source: J Anim Sci Biotechnol. 2022 Jan 5;12:126. doi: 10.1186/s40104-021-00647-2 (PMC8729039; doi:10.1186/s40104-021-00647-2)
Supplement: Supplementary file 1 — Additional file 1: Supplementary materials. The flow cytometry density map of peripheral blood at the end of 5th week (27 weeks old, n = 6). Supplementary materials: the flow cytometry density map of peripheral blood at the end of 10th week (32 weeks old, n = 8). [file 40104_2021_647_MOESM1_ESM.docx]

**Supplementary materials: the flow cytometry density map of peripheral blood at the end of 5^th^ week (27 weeks old, *n* = 6)**


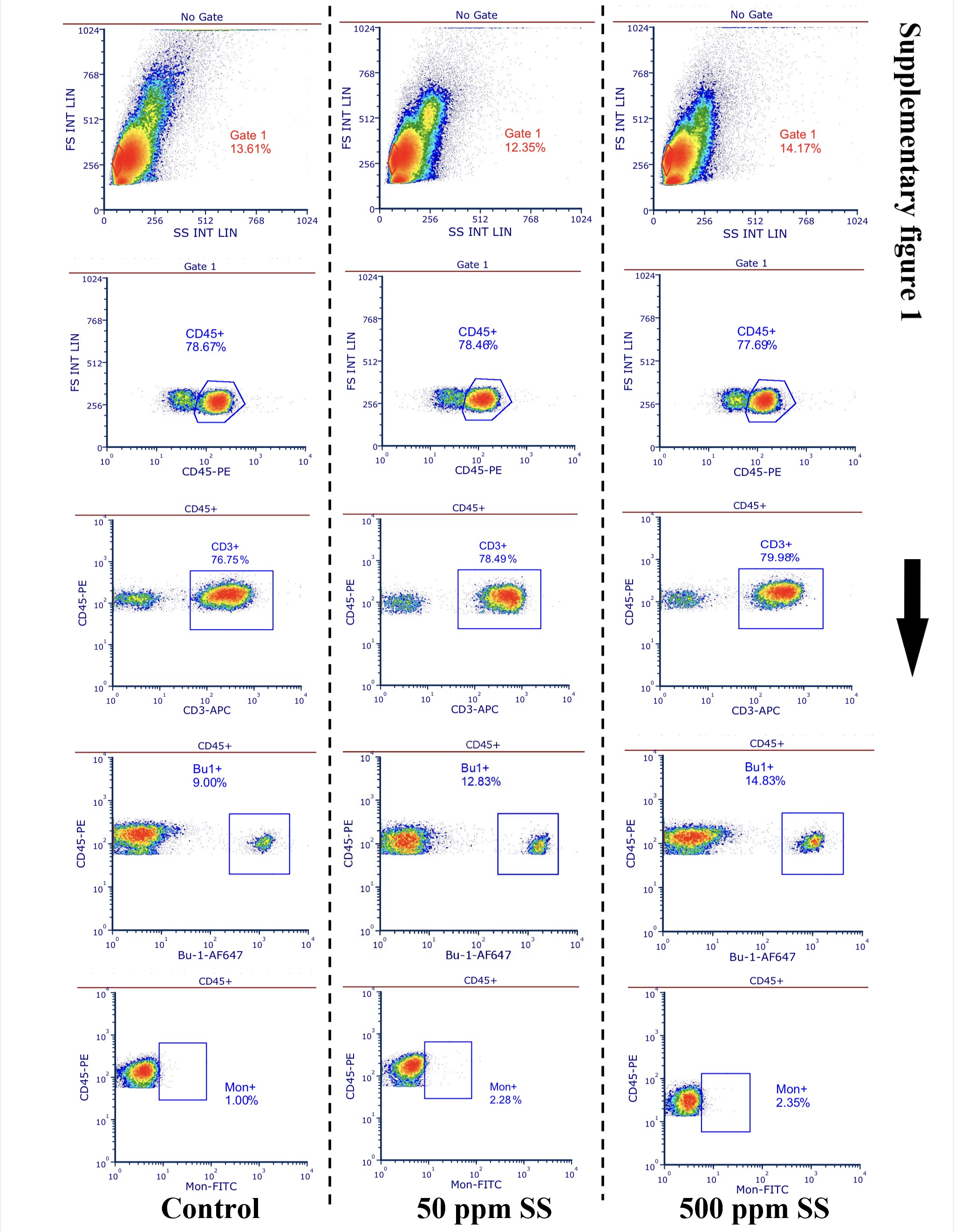


**Supplementary materials: the flow cytometry density map of peripheral blood at the end of 10^th^ week (32 weeks old, *n* = 8)**

**
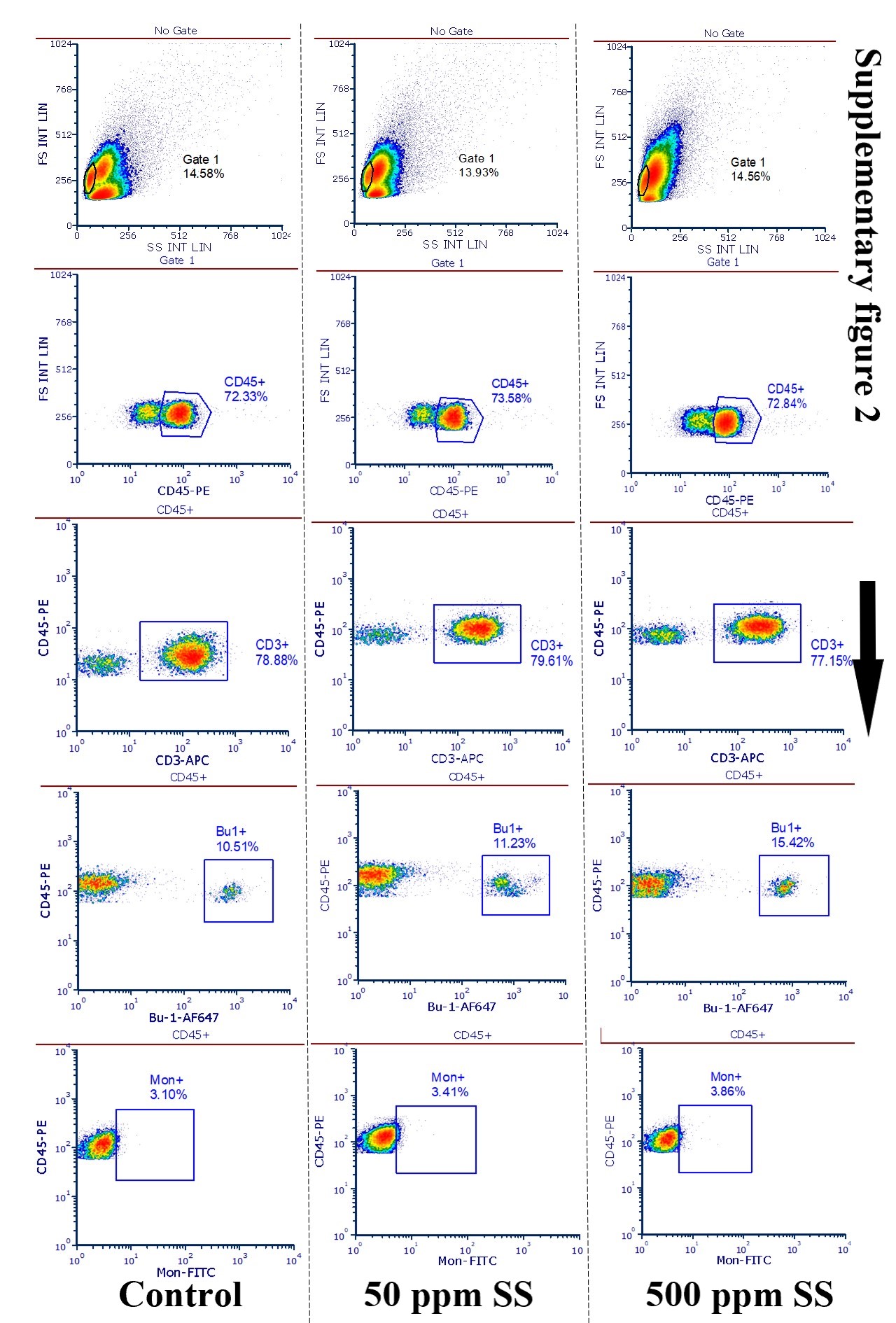
**
